# The relationship between headache and chronic musculoskeletal complaints: an 11-year follow-up in the The Nord-Trøndelag Health Study (HUNT)

**DOI:** 10.1186/1129-2377-14-S1-P12

**Published:** 2013-02-21

**Authors:** LJ Stovner, K Hagen, M Linde, TJ Steiner, JA Zwart

**Affiliations:** 1 Department of Neuroscience, Norwegian university of Science and Technology, UK; 2 Department of Neuroscience, Norwegian university of Science and Technology, Norway; 3 Oslo University Hospital, Norway

## Background and aim

Chronic daily headache (CDH) and chronic musculoskeletal complaints (CMSCs) are associated disorders, but whether there is a causal relationship between them is unclear.The aim of the study was to determine whether CMSCs are associated with the subsequent development of CDH and vice versa.

## Methods

This longitudinal population-based cohort study used data from two consecutive surveys in the Nord-Trøndelag Health Study (HUNT 2 and 3) performed in 1995-1997 and 2006-2008. Amongst the 51 383 participants aged ≥20 years at baseline, 41 766 were eligible approximately 11 years later. Of these, 26 197 (63%) completed the questions regarding headache and CMSCs in HUNT 3.

## Results

A bidirectional relationship was found between headache and CMSCs. In the multivariate analyses adjusting for potential confounders, a nearly twofold risk (OR 1.8; 95% CI 1.5-2.3) for developing CDH was found for those with CMSCs at baseline. Vice versa, a similarly elevated risk of CMSCs (OR 1.8; 95% CI 1.2-2.6), and even higher risk of chronic widespread MSCs (OR 2.7; 95% CI 1.6-4.7), was found at follow-up amongst those with CDH at baseline.

## Conclusion

Chronic musculoskeletal complaints predispose to CDH and CDH predisposes to CMSCs 11 years later. This may have relevance to understanding the pathophysiology of these disorders. CMSCs should be treated not only to relieve them but also to prevent the development of CDH, and vice versa.

